# Filters and Filtering

**Published:** 1872-07

**Authors:** 


### SELECTIONS

### Filters and Filtering.

Water, wine, spirits, jelly, syrup, tinctures and a great variety of other fiuids, hot and cold, often contain substances which should be separated, in order to render the fluid clear and bright. As regards water filtering, it has become pretty general; but in domestic life there are fluids, such as wine, liquid jelly, syrup, etc., which are required to be made "clear" before they are put on the table. There are three kinds of filters—sponge for watery liquids, cotton for spirituous liquors, and wool for gelatinous fluids and oils. In every well-appointed kitchen there are tin or porcelain funnels. For filtering watery fluids it is only necessary to insert, in the choke of the funnel, a V shaped piece of fine sponge. All such liquids, on being put into the funnel, will pass through the sponge, and become quite clear. When this effect ceases, the sponge must be removed and well cleansed. Vinous fluids are best cleared by filtering through a cone of white blotting-paper, shaped by folding a square piece of paper from corner to corner, then folding the triangle into half its size and opening the folds; it will fit any funnel, which will act as a support the paper.

Wines, etc., poured into this, will run through perfectly bright. In some cases where the wine is only a little thick from lees, cork, or other mechanically suspended substance, it can be made clear by filtering through a wad of white cotton, put in the choke of the funnel; and when this answers it is much quicker than the paper filter. For jelly and oil, wool alone is the proper medium for filtering. The felted wool jelly bag is pretty well known as the best means of clearing calves'-foot jelly, and it also answers for olive and other oils. These bags, however, are too expensive to be generally used; hence they are rarely seen in kitchens. A good substitute for the wool bag is the colander, on the inside of which a new flannell should be fitted, made of double stuff. A wad of white knitting wool, put in the choke of the funnel, will do to filter any small portion of such fluids. Many a good glass of port wine has been wasted for the want of a penny paper filter.

